# Botulinum Neurotoxin for the Treatment of Neuropathic Pain

**DOI:** 10.3389/fneur.2020.00716

**Published:** 2020-08-11

**Authors:** Gabriella Egeo, Luisa Fofi, Piero Barbanti

**Affiliations:** ^1^Headache and Pain Unit, Department of Neurological, Motor and Sensorial Sciences, IRCCS San Raffaele Pisana, Rome, Italy; ^2^San Raffaele University, Rome, Italy

**Keywords:** botulinum toxin, neuropathic pain, pain treatments, visual analog scale, disability

## Abstract

Botulinum neurotoxin is widely used for the treatment of central and peripherical neurological conditions. Initially used to treat strabismus, over the years its use has been expanded also to spasticity and other neurological disorders. This review summarizes the evidence from the published literature regarding its effect on neuropathic pain. Almost all investigations were performed using onabotulinum toxin type A (BoNT/A). Most studies provided positive results, even though toxin formulation, dose, dilution, injection techniques, and sites are heterogeneous across studies. Future larger, high-quality, specifically designed clinical trials are warranted to confirm botulinum neurotoxin efficacy in neuropathic pain.

## Introduction

Neuropathic pain (NP) is a pain caused by a lesion or a disease affecting the somatosensory nervous system and encompasses common neurological pain syndromes such as trigeminal neuralgia (TN), postherpetic neuralgia (PHN), diabetic neuropathic pain (DN), and postsurgical neuralgia.

NP is caused by pathological changes involving the peripheral (nerves, plexus, roots, and sensitive ganglia) and the central nervous system (CNS). The pathologies responsible for tissue specific symptoms of NP comprise viral infections (e.g., herpes simplex, varicella zoster, and human immunodeficiency virus), metabolic disorders with mitochondrial dysfunctions (e.g., diabetes), stroke, mechanical injuries to the CNS or peripheral nerves (1, 2), and toxic effects, above all anti-neoplastic compounds (e.g., oxaliplatin, vincristine) (3).

Nociceptor activation is one of the most relevant NP peripheral mechanism, causing abnormal neuronal hyperexcitability, hyperalgesia, and allodynia (4–6).

Nociceptors consist of free nerve endings related to unmyelinated C-fibers and small-myelinated Aδ-fibers; they are activated by different mechanical, thermal, and chemical stimuli and a variety of endogenous substances [e.g., substance P (SP), bradykinin, serotonin, calcitonin gene-related peptide (CGRP), prostaglandins, excitatory amino acids histamine, growth factors, and proinflammatory cytokines] (4–6). Increased nociceptor excitability due to nerve injury causes glutamate-mediated pronociceptive activation and a decrease in inhibitory influences. Primary nociceptive Aδ- and C-fibers terminate at two distinct types of spinal second-order neurons, i.e., spinal neurons projecting to higher neuronal structures, and spinal interneurons modulating synaptic transmission in the dorsal horn where resident microglial activation plays also a key role (7). Glutamate participates in the transmission of nociceptive inputs from the periphery to the brain by binding to α-amino-3-hydroxy-5-methyl-4-isoxazolepropionic acid (AMPA), N-Methyl-D-Aspartate (NMDA), and metabotrobic (mGluR) receptors. The glutamate pathway mediates basic responses to nociceptive stimuli and contributes to the spinal dorsal horn hyperexcitability, manifesting with synaptic plasticity, and long-term potentiation (6–8).

The activity of second-order neurons is modulated by the descending brainstem inhibitory noradrenergic, serotonergic, and opiatergic pathways, spinal GABAergic and glycine inhibitory inputs, as well as by the cannabinoid system (8–10).

Current pharmacological and non-pharmacological treatment of NP is still unsatisfactory (9, 11, 12). Besides lidocaine, capsaicin, antidepressants, anticonvulsants, and opioids, botulinum toxin (BoNT) has more recently emerged as a promising NP therapeutic strategy (13, 14). The first evidence of BoNT efficacy in NP in humans dates back to 2001 when Freund and Schwartz (15) described seven patients with postherpetic neuralgia (PHN) treated for >6 months with subcutaneous BoNT injections at 38th Interagency Botulism Research Coordinating Committee Meeting (15). Currently, the use of BoNT is considered for NP whenever common pharmacological agents have been ineffective (16).

BoNT is a potent neurotoxin produced by Clostridium botulinum, which blocks acetylcholine release at neuromuscular junctions causing muscle relaxation. The mechanism of action of BoNT in NP is related to the inhibition of the release of neurotransmitters and neuropeptides involved in pain mechanisms and inflammation (substance P, CGRP, glutamate) (16, 17). Moreover, BoNT reduces the activity of the transient receptor potential vanilloid 1 ion channels (TRPV1), involved in the transduction of noxious stimuli (18, 19). Two different BoNT serotypes are used: Botulinumtoxin A (BoNT/A)—encompassing onabotulinumtoxinA (A/Ona, BOTOX® Allergan), abobotulinumtoxinA (A/Abo, Dysport® Ipsen), and incobotulinumtoxinA (A/Inco, Xeomin® Merz)—and Botulinumtoxin B (BoNT/B) i.e., rimabotulinumtoxinB (B/Rima, Myobloc®/Neurobloc® ElanPharmaceuticals). These toxins differ for complexity, purity, potency, dosing, and immunogenicity. Most studies in NP have been conducted using BoNT/A.

The aim of the present paper is to systematically review the evidence on BoNT usefulness in the management of NP, to highlight scientific certainties and doubts, addressing research progresses, and suggesting directions for future investigations.

## Materials and Methods

### Search Strategy and Criteria for Selecting Articles

**Figure 1:**
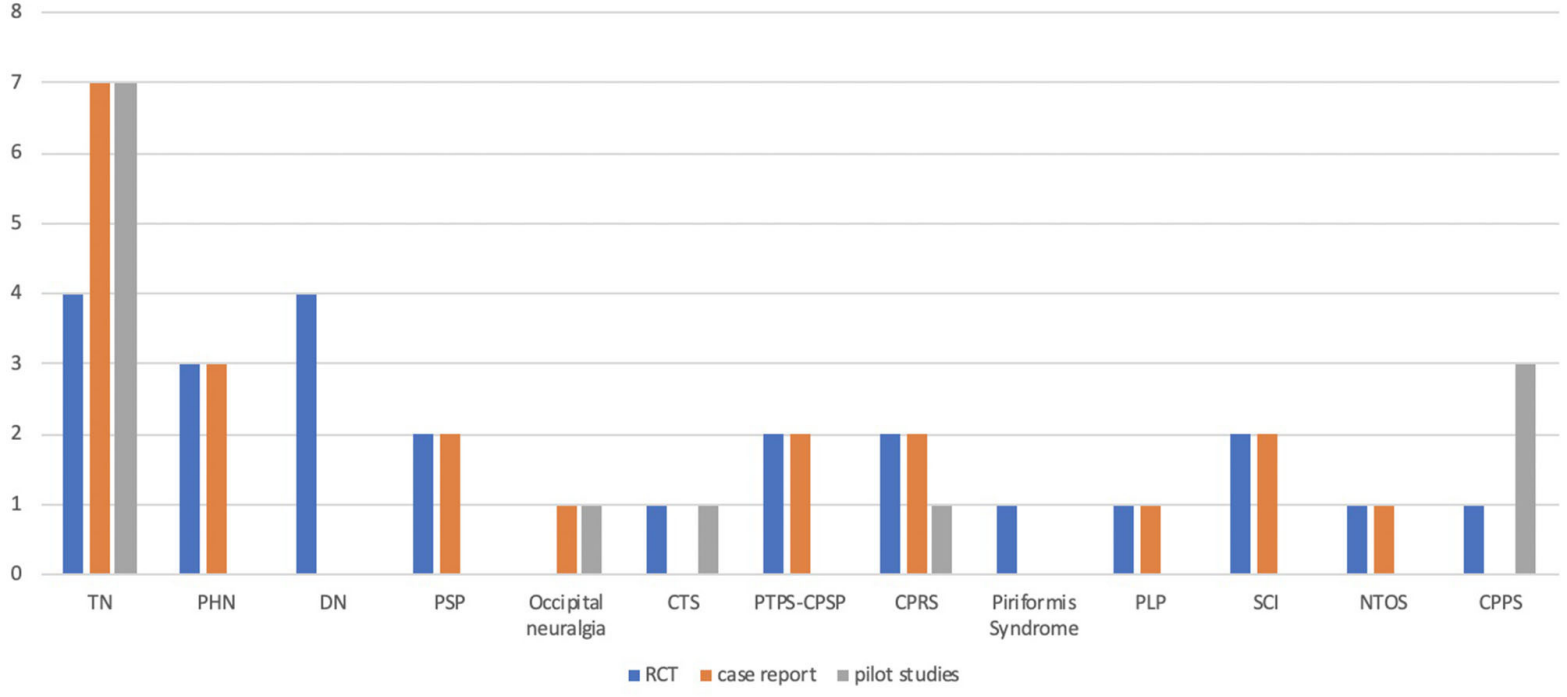
Studies assessing the efficacy of botulinum toxin in different types of neuropathic pain. TN: trigeminal neuralgia; PHN: post-herpetic neuralgia; DN: diabetic neuropathy; PSP: post-stroke pain; CTS: carpal tunnel syndrome; PTPS: post-thoracotomy pain syndrome; CPSP: chronic post-surgical pain; CPRS: complex regional pain syndrome; PLP: phantom limb pain; SCI: spinal cord injury; NTOS: neurogenic thoracic outlet syndrome; CPPS: Chronic pelvic pain syndrome.

We searched the electronic database MEDLINE, PubMed, and the Cochrane Database for published papers and extracted data for (1) pain, (2) neuropathic pain, (3) botox, (4) botulinum toxin, (5) neuralgia, and (6) neuropathy. We considered randomized controlled trials (RCT), open label (OL) studies, retrospective/prospective case-control (CC) studies, case reports (CR), and case-series (CS) on adult patients with neuropathic pain. Our search also included meta-analyses with no language restrictions and all the titles and abstracts identified by the search were evaluated for eligibility (Figure 1).

We considered all the articles on human studies providing abstract and full-text published in English language, regardless the year of publication.

We also searched clinical trials on BoNT and NP on www.clinicaltrials.gov.

The search and selection of the articles were made independently by two evaluators (GE, LF) and then discussed with the third author (PB).

## Results: BoNT in Neuropathic Pain

**Figure 2:**
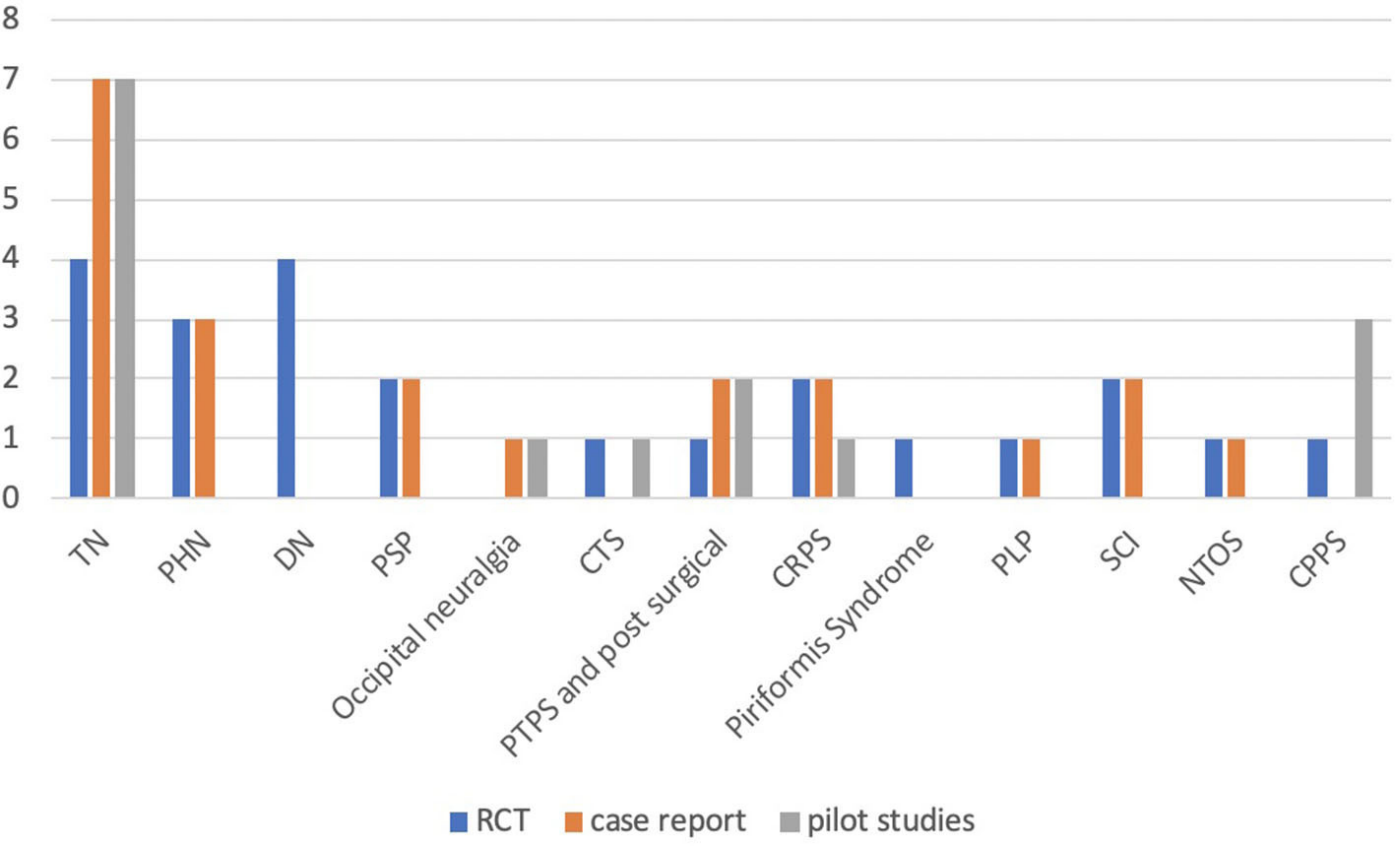
Studies assessing the efficacy of botulinum toxin in different types of neuropathic pain. TN: trigeminal neuralgia; PHN: post-herpetic neuralgia; DN: diabetic neuropathy; PSP: post-stroke pain; occipital neuralgia; CTS: carpal tunnel syndrome; PTPS: post-thoracotomy pain syndrome; post-surgical pain; CRPS: complex regional pain syndrome; PLP: phantom limb pain; SCI: spinal cord injury; NTOS: neurogenic thoracic outlet syndrome; CPPS: Chronic pelvic pain syndrome.

Among the numerous pharmacological studies on BoNT for NP treatment in adults, we identified 22 RCTs, 20 CR, and 10 OL studies including a total of 1,543 patients. Eighteen studies focused on the effect of botulinum toxin in TN, nine in traumatic, compressive and post-surgical causes of NP, six in PHN, five in complex regional pain syndrome (CRPS), four in post-stroke pain (PSP), four in spinal cord injury (SCI), three in painful diabetic neuropathy (PDN), two in occipital neuralgia, two in phantom limb pain (PLP), two in neurogenic thoracic outlet syndrome (NTOS), and four in chronic pelvic pain syndrome (CPPS) (see Figure 2). The search on www.clinicaltrials.gov documented 17 experimental trials: nine completed, four terminated, two ongoing, and one withdrawn. One study actually focused on migraine and was therefore exclude. Complete results are available only for one of the above trials included in our review.

### Trigeminal Neuralgia

TN is the most common and disabling cranial neuralgia in adults and is characterized by a unilateral, abrupt, brief electric shock-like pain, limited to the distribution of one or more divisions of the trigeminal nerve, typically triggered by innocuous stimuli (20). TN is etiologically classified as *idiopathic* (without any reliable organic substrate), *classic* (due to a neurovascular conflict between an anomalous vessel and the trigeminal root close to its entry into the pons), and *secondary* (due to major neurologic diseases, such as multiple sclerosis or tumors at the cerebellopontine angle) (21).

Indisputably, carbamazepine (400–1,200 mg/day) and oxcarbazepine (900–1,800 mg/ day) represent the first-choice TN medical treatment (22). However, even though they are effective in 80% of patients, their clinical benefit may decrease over time and their use is frequently associated to significant side effects (drowsiness, nausea, dizziness, ataxia, hyponatremia, and liver enzymes elevation) (23). Neurosurgical procedures (such as microvascular decompression and radio-surgical treatment)—considered for refractory cases—induce clinical benefit in almost 60–90% of cases but may be followed by complications or pain recurrence (23, 24).

Diverse investigations provided encouraging data on the efficacy of onabotulinum toxin type A (BoNT/A) in reducing pain severity and attack frequency in TN (25, 26). It's worth mentioning, however, that these studies are quite heterogeneous in terms of BoNT/A dose, dilution, route of administration, number/sites of injection and needle type used and include CR (*n* = 7) (27–33), OL studies (*n* = 8) (34–41), and RCTs (*n* = 4) (42–45) (Table 1).

**Table 1:** Studies on the use of botulinum toxin in TN.

**References**	**Study type**	**Blinding**	**Comparator**	**Pts n^**°**^**	**Injection route/site**	Toxin type*	**Dose (U)**	**Major findings**	**AEs**
(27)	Case report	na	na	1	IM	Botulinum toxin type A-Botox®	16 × 8	VAS reduction score from 82 (baseline) to 45	-
(28)	Case report	na	na	1	IM	Botulinum toxin type-A	7.5 × 2	More than 90% relief over 2 months with consequent analgesic overuse cessation.	-
(29)	Case report	na	na	1	SC	Botulinum toxin type A-Botox®	100	Complete pain relief at the right external nasal region on the second day after the injection. Partial relief at the mental region. Recurrence after 5 months	Facial paralysis
(31)	Case report	na	na	1	ID, SC	Onabotulinumtoxin type A	100	Improvement after 15 days followed by total pain relief. Pain disappearance at month 28	Local injection site swelling and slight distal eye- brow ptosis
(32)	Case report	na	na	2	SM	Onabotulinum toxin A-Botox®	100	*Case 1:* after 3 months, VAS score reduction from 5 to 2*; Case 2*: after 3 months, VAS score reduction from 10 to 3.	Dryness in the injection area, facial asymmetry
(33)	Case report	na	na	1	First treatment: SM, IM	Botulinum toxin type A- HengLi® Botox, Lanzhou, Gansu, China	50	VAS score reduction from 5–8 to 3–5 after 1 week; pain free after 2 weeks	None
(34)	OL	s	na	8	SC	Onabotulinum toxin A-Botox®	100	VAS score reduction	None
(35)	OL	s	na	13	SC	Botulinum-A neurotoxin	NS	Significant pain reduction at day 10, symptom free at day 20, > 50% reduction in preventive medication, multiple medications reverted to monotherapy	None
(36)	OL	s	na	20	SC	Botulinum toxin type A-Botox®	20–50	Significant reduction in VAS score (8.83 to 4.08) and in n° of paroxysms (from 23.42 to 8.67).	-
(37)	OL	s	na	15	SC	BoNT/A	50	Significant reduction in VAS score and attack frequency at 1 week, 1 month, and 6 months after injection (*P* = 0.001). 7 patients became pain free	Transient paresis of the buccal branch of the facial nerve in 3 patients
(38)	OL	s	na	88	SC	Botulinum toxin type A- HengLi® Botox, Lanzhou, Gansu, China	25–170	Effective <1st month in 81 pts and at 2nd month in all subjects. The therapeutic effect decreased after the 3rd month	Local swelling at injection in 3 pts, facial paralysis in 10 pts
(39)	OL	s	na	100	ID, SM	Botulinum toxin type A- HengLi® Botox, Lanzhou, Gansu, China	70–140	Significant VAS score reduction; comparable efficacy and side effects between single and repeated doses.	“Mild, moderate side effects”
(40)	OL	s	na	27	Maxillary and mandibular root	Onabotulinum toxin A-Botox®	100	At month 6 significant reduction in VAS score (from 9.7 to 1.6) and attack frequency (from 217.7 to 55.15); 44.4% of the patients were pain-free	“Mild, moderate side effects
(41)	OL	s	na	43	ID, SM	Botulinum toxin type A- HengLi® Botox, Lanzhou, Gansu, China	30–200	Significant VAS score reduction: in older patients from 8.5 to 4.5 after 1 month, in younger patients from 8.0 to 5.0	Whole-body mild discomfort, left eye ptosis + slight oral deviation/drooling (2 pts); facial paralysis (2 pts)
(42)	RCT	d	Placebo	42	ID, SM	BoNT/A	75	Significant reduction in VAS score and attack frequency after 2 weeks	Facial asymmetry, facial oedema
(44)	RCT	d	Placebo	36	SM, IM	Botulinum toxin type A-Botox®	50	Significant VAS score reduction after 2 months (*p* = 0.07) and after 3 months (*p* = 0.01)	Hematoma, slight facial asymmetry
(43)	RCT	s	Placebo	20	SM, IM	Botulinum toxin type A-Botox®	40–60	Significant reduction in VAS score and number of weekly acute medications and increase in QoL functioning scale	Transient facial asymmetry, hematoma, itching and pain at the site of injection
(45)	RCT	d	Placebo	84	ID, SM	BoNT/A	25 or 75	No difference in short term efficacy with low or high dose	Transient facial asymmetry, oedema at the site of injection

ITN, idiopathic trigeminal neuralgia; TN, trigeminal neuralgia; s, single blind arm; d, double blind; na, not applicable; ^*^term as originally reported in the study; U, units; IM, intramuscular; SC, subcutaneous; ID, intradermal; SM, submucosal; RCT, randomized controlled trials; OL, open label; BoTN/A, onabotulinum toxin type A; VAS, visual analog scale; -, not reported.

In one RCT, 42 patients affected by classical TN were randomized to multiple intradermal and/or submucosal injections of BoNT/A 75U (22 pts) or saline (20 pts) in the skin and/or mucosa of affected pain areas. BoNT/A significantly reduced pain intensity at week 2 and pain attack frequency at week 1 compared to the placebo (68.2 vs. 15.0%; *p* < 0.01), showing sustained efficacy and good tolerability (42).

BoNT/A (100 U) has been demonstrated to be effective also in intractable TN in a randomized, single-blinded, placebo-controlled study on 20 patients, significantly reducing pain intensity (6.5 vs. 0.3; *p* < 0.0001) and acute medication intake and increasing quality of life (QoL) functioning scale at week 12. Each point injection, detected using a “follow the pain” method, received BoNT/A (5 U) or 0.1 ml placebo subcutaneously. In patients with mandibular root involvement, a larger toxin dose was injected posteriorly in the masseter to avoid undesired cosmetic effects (43).

Similarly, BoNT/A (50 U) outperformed placebo in pain severity reduction at 3 months post-dosing (VAS 4.75 vs. 6.94; *p* = 0.01) in a RCT on 36 patients (20 randomized to active and 16 to placebo) affected by idiopathic TN. Injections were delivered subcutaneously in the affected area and also in the masseter muscle in patients with the involvement of the third branch of the trigeminal nerve (44).

The efficacy of BoNT/A in the treatment of classical TN is not dose-dependent. Zang et al. (45) randomized 84 patients to placebo (*n* = 28), BoNT/A 25U (*n* = 27), or BoNT/A 75U (*n* = 29) and found that both toxin doses were equally significantly more effective than placebo in reducing VAS scores as early as week 1, showing higher response rates at week 8 (70.4 and 86.2%, respectively, vs. 32.1%; *p* < 0.017). The proportion of patients reporting their pain symptoms as “much improved” or “very much improved” at Patient Global Impression of Change was comparable at both 25U (66.7%) and 75U (75.9%) doses and clearly superior to placebo (32.1%).

A recent meta-analysis of 4 RCTs (42–45) including 178 patients (BoNT/A: 99; placebo: 79) revealed a significant superiority of BoNT/A in reducing pain intensity as measured by VAS total score (RR 2.87, *p* < 0.0001) and frequency of attacks (*p* < 0.0001), documenting a benefit duration up to 3 months and mild–moderate and self-limiting adverse events (46). BoNT/A proved effective also in TN persisting after microvascular decompression surgery (29), in symptomatic TN due to exostosis in Meckel's cave (33) and in refractory ITN (40).

To sum up, BoNT/A may represent a useful therapeutic tool in the clinical management of TN. However, the low quality of evidence has led the recent European Academy of Neurology guidelines to limit the use of BoNT/A to a medium-term treatment “in some selected cases” (Table 1) (47).

### Post-herpetic Neuralgia

Herpes zoster results from reactivation of varicella zoster virus which lies dormant in sensory dorsal roots, cranial nerves, and autonomic ganglia. It presents as a painful maculopapular or vesicular rash in a dermatomal distribution, most commonly in thoracic and cranial distributions. The PHN is its most common complication, occurring in 10–50% of patients (48), and it can persist for weeks or years after regression of the rash, impairing patients' quality of life (49).

BoNT/A may represent an effective therapeutic option for PHN. Some case-reports documented the efficacy of the toxin in patients with PHN refractory to conventional treatments in term of VAS score reduction at a mean dose of 100 U, with an analgesic effect duration ranging from 52 to 64 days and a good tolerability (50–52).

The encouraging results of this small series have been confirmed by three RCTs and a recent meta-analysis. In one RCT with active comparator, 60 patients affected by PHN in different cutaneous areas were randomized to receive BoNT/A (20 pts), lidocaine (20 pts), or placebo (20 pts). The volumes of administration varied according to the area of tactile allodynia, but fewer than 40 mL volumes (200 units for the maximum BoNT/A dose) were used. BoNT/A reduced pain more effectively than both lidocaine and placebo at day 7 and after 3 months compared to baseline (*p* < 0.01). The improvement of sleep time in the BoNT/A group was also significantly greater compared with the other groups (*P* < 0.01) (52, 53).

A long-lasting BoNT/A therapeutic effect has been confirmed in a RCT on 30 adults with PHN in which only the 13 subjects randomized to BoNT/A achieved a >50% reduction in VAS score (NNT=1.2, 95% CI, 2–1; ARR=0.87, 95% CI, 055–096; *P* < 0.001). Notably, BoNT/A improvement in pain and sleep scores persisted for 16 weeks (54).

In a RCT on 68 patients affected by a miscellany of diverse peripheral neuropathic pain syndromes (post-traumatic or postsurgical pain, polyneuropathy, postherpetic neuralgia), 34 subjects were randomized to receive two subcutaneous administrations of BoNT/A (up to 300 U) or placebo, 12 weeks apart. Two successive BoNT/A administrations significantly decreased (*p* < 0.0001) the mean pain intensity over 24 weeks after the first treatment administration compared with placebo. The study, which included six subjects with PHN, confirmed the role of BoNT/A in reducing pain severity, evidencing that it was particularly efficacious in participants with preserved nociceptive input. Moreover, the authors suggested that at least two administrations of BoNT/A might be necessary in non-responders before deciding to withdrawing the treatment (55).

Despite BoNT/A efficacy in both chronic TN and PHN (56), herpes zoster has been described as a complication of BoNT/A administration in a 72-year-old woman affected by chronic migraine who developed ophthalmicus herpes zoster 5 days after treatment, probably due to local stress reaction following tissue injury inducing VZV reactivation (57).

In conclusion, the efficacy and safety of BoNT/A in the treatment of PHN is supported by scientific evidence (Table 2), but studies on larger populations are needed.

**Table 2:** Studies on the use of botulinum toxin in PHN.

**References**	**Study type**	**Blinding**	**Comparator**	**Pts n°**	**Injection route/site**	Toxin type*	**Dose (U)**	**Major findings**	**AEs**
(50)	Case report	Na	na	1	SC	Botulinum toxin type A- HengLi® Botox, Lanzhou, Gansu, China	100	VAS score reduction from 10 to 1 after 2 days, lasting 52 days	-
(51)	Case report	na	na	1	ID	BoNT/A	-	VAS score reduction lasting 2 months	-
(52)	Case report	na	na	3	SC	Botulinum toxin type A-Botox®	100	VAS score reduction from 8.3 to 2 after 2 weeks	Temporary erythema
(53)	RCT	d	Placebo	60 (20 treated)	SC	BoNT/A	200	Significant reduction of overall symptoms severity (pain, opioid use, sleep interference); marked improvement in quality of life.	-
(54)	RCT	d	Placebo	30 (15 treated)	SC	BoNT/A	200	Significant reduction in VAS and sleep scores at week 2; the effect lasted 16 weeks.	Pain during injections
(55)	RCT	d	Placebo	6	SC	Botulinum toxin type A-Botox®	up to 60 × 5	Significant VAS score reduction	-

PHN, post-herpetic neuralga; RCT, randomized controlled trials; s, single blind arm; d, double blind; na, not applicable; IM, intramuscular; SC, subcutaneous; ID, intradermal; SM, submucosal; *term as originally reported in the study; BoTN/A, onabotulinum toxin type A; U, units; VAS, visual analog scale; -, not reported.

### Diabetic Neuropathy

DN is a common debilitating complication of diabetes. About a third of patients develop painful diabetic neuropathy (PDN) (58, 59). As current pharmacological treatments are not always effective, BoNT/A has been investigated for pain control in PDN in some RTCs.

Intradermal BoNT/A administration in 18 patients (50 U in the dorsum of the foot for a total of 12 sites at the dose of 4 U for each injection point) induced a significant reduction in VAS score at weeks 1, 4, 8, and 12 compared to placebo with a reduction of VAS ≥3 within 3 months after injection in 44% of patients receiving the active drug. A transient sleep quality improvement was also described (60). Using the same study protocol, Chen et al. (61) reported that BoNT/A may also be beneficial in reducing tactile and mechanical pain threshold in PDN (61). Intradermal BoNT/A at the dose of 8–10 U per injection site (total dose = 100 U) induced a significant (*p* = 0.05) reduction in neuropathic pain scale (NPS) scores for all items—except cold sensation—in VAS (*p* = 0.01) and DN4 scores (*p* < 0.05) compared to placebo in a study performed on 40 patients affected by PDN aged <70 years. One third of patients in the treatment group showed bilateral pain reduction 3 weeks after injection (62).

The use of BoNT/A in DN is promising although the studies are scarce and carried out on small populations (60–63). Larger RTCs are needed.

### Post-stroke Pain (PSP)

PSP is a heterogeneous clinical entity caused by neuropathic and nociceptive mechanisms, which affects from 10 to 70% of patients with stroke and includes central PSP, pain related to spasticity, muscle-skeletal pain, complex regional pain syndrome, and post-stroke headache (64, 65).

PSP is one of the factors contributing to patients' disability and interferes with daily activities, sleep, walking, physiotherapy, greatly affecting their quality of life.

The efficacy of BoNT/A in central PSP control has been investigated (Table 3) (66–69). A prospective RCT performed on 37 patients failed to demonstrate any BoNT/A efficacy on PSP (68). Conversely, a larger RCT on post-stroke spasticity on 273 patients—mostly complaining of PSP (74.3%)—randomized to BoNT/A plus standard care showed a significantly greater reduction in pain and in pain interference with work (*p* < 0.05) compared to patients treated with placebo plus standard care (Table 4) (69).

**Table 3:** Studies on the use of botulinum toxin in DN.

**References**	**Study type**	**Blinding**	**Comparator**	**Pts n°**	**Injection route/site**	Toxin type*	**Dose (U)**	**Major findings**	**AEs**
(60)	RCT	d	Placebo	20	ID	Botulinum toxin type A	50	Significant VAS reduction (*P* < 0.05)	Local skin infection
(61)	RCT	d	Placebo	20	ID	Onabotulinum toxin A	50	Significant decrease in tactile threshold and pain threshold (*P* < 0.05)	-
(62)	RCT	d	Placebo	40	ID	Botulinum toxin type A-Dysport®-Ipsen, UK	100	Reduction in NPS (*P* = 0.05) and DN4 scores (*P* < 0.05). Pain freedom in 30% of patients (*p* = 0.01)	-

DN, diabetics neuropathy; RCT, randomized controlled trials; d, double; ID, intradermal; *term as originally reported in the study; U, units; VAS, visual analog scale; NPS, neuropathy pain scale; -, not reported.

**Table 4:** Studies on the use of botulinum toxin in PSP.

**References**	**Study type**	**Blinding**	**Comparator**	**Pts n°**	**Injection route/site**	Toxin type*	**Dose (U)**	**Major findings**	**AEs**
(67)	Case report	na	na	1	IM	Onabotulinum toxin type A-Botox®	25, 75, and 100	Pain reduction after 2 days; spasticity improvement after 1 week	-
(66)	Case report	na	na	2	IM	Botulinum toxin type A	200	NRS reduction for more than 3 months	-
(69)	RCT	d	Placebo+SC	273 (139 BoNT/A + SC vs. 134 Placebo + SC)	IM	Onabotulinum toxin type A-Botox®	As needed	Reduction in VAS and pain interference with work (P < 0.05)	-
(69)	RCT	d	Placebo	37 (21 BoNT/A vs. 16 Placebo)	IM	Onabotulinum toxin type A-Botox®	140, 200	No significant differences between the groups found for any of the daily pain ratings	-

PSP, Post-stroke pain; RCT, randomized controlled trials; d, double; na, not applicable; SC, Standard care; IM, intramuscular; *term as originally reported in the study; U, units; VAS, visual analog scale; -, not reported.

The usefulness of BoNT/A in the management of PSP deserves to be investigated in further *ad hoc* designed RTCs.

### Occipital Neuralgia

Occipital neuralgia is a unilateral or bilateral radiating pain in the posterior part of the scalp in the distribution of the greater, lesser, and/or third occipital nerves (20). The causes of occipital neuralgia include irritation or injury to the divisions of the occipital nerve, its focal entrapment and myofascial spasm. Persistent occipital neuralgia can produce severe headaches that are difficult to control by conservative or surgical approaches. The occipital nerve blocks using BoNT/A at the dose of 50 U for each block provided a meaningful reduction in pain intensity and disability in five out of six patients in patients who had failed prior oral therapies or traditional nerve blocks in a case series study (70). BoNT/A, at the same dose, improved the sharp/shooting type of pain associated with occipital neuralgia in a pilot study on six patients, inducing also a significant improvement of headache-specific quality of life (*p* = 0.0315) (Table 5) (71).

**Table 5:** Studies on the use of botulinum toxin in Occipital Neuralgia.

**References**	**Study type**	**Blinding**	**Comparator**	**Pts n°**	**Injection route/site**	Toxin type*	**Dose (U)**	**Major findings**	**AEs**
(70)	Cases series	na	na	6	Occipital nerve block	Onabotulinum toxin type A-Botox®	100 (50 for each block)	Significant reduction in pain VAS scores and improvement in PDI	-
(71)	PS	s	na	6	Occipital nerve block	Onabotulinum toxin type A-Botox®	100 for each block	Improvement in the sharp/shooting type of pain most commonly associated with occipital neuralgia	-

PS, prospective study; s, single; na, not applicable; ID, intradermal; *term as originally reported in the study; U, units; VAS, visual analog scale; PDI, pain disability index; -, not reported.

### Carpal Tunnel Syndrome (CTS)

ARCT with botulinum toxin type B (BoNT/B) in 20 outpatients affected by CTS did not confirm the positive findings reported in an open label trial on five women (Table 6) (72, 73).

**Table 6:** Studies on the use of botulinum toxin in CTS.

**References**	**Study type**	**Blinding**	**Comparator**	**Pts n°**	**Injection route/site**	Toxin type*	**Dose (U)**	**Major findings**	**AEs**
(72)	PS	s	na	5	Intracarpal	Botulinum toxin type A- Dysport®, Beaufour Ipsen, UK	60	Not superior to placebo (*p* = 0.2)	Local weakness and discomfort
(73)	RCT	d	placebo	20	Intracarpal	Botulinum toxin type B	30 for sides	Not superior to placebo	-

CTS, carpal tunnel syndrome; PS, prospective study; RCT, randomized controlled trials; s, single; d, double; na, not applicable; *term as originally reported in the study; U, units; -, not reported.

### Post-thoracotomy Pain Syndrome (PTPS) and Chronic Post-surgical Pain

PTPS is a traumatic neuropathy that can affect as many as 50% of patients undergoing thoracotomy, is often refractory to conservative management and may require multiple analgesics for adequate pain control. BoNT/A may represent an alternative or adjunct treatment to improve symptom management in patients with PTPS (Table 7) (79). Two case reports documented the efficacy of BoNT/A (total dose: 50–100 U, along the scar) in inducing a significant and prolonged pain reduction in patients affected by PTPS with multiple prior therapeutic failures (74, 75).

**Table 7:** Studies on the use of botulinum toxin in post-surgical syndrome.

**References**	**Cause of pain**	**Study type**	**Blinding**	**Comparator**	**Pts n°**	**Injection route/site**	Toxin type*	**Dose (U)**	**Major findings**	**AEs**
(74)	Post-thoracotomy	Case report	na	na	1	SC	Onabotulinum toxin type A-Botox®	2.5 for site	50% VAS score improvement, sustained up to week 12	-
(75)	Post-thoracotomy	Case report	na	na	1	SC	Botulinum toxin type A	50	Significant reduction of pain at day 4 sustained up to month 4	-
(76)	Post-surgical and post radiation therapy	PS	s	na	25	IM, SC	Incobotulinum toxin A	100	Significant improvement in VAS score and patients satisfaction	2 skin reactions
(77)	Post-mastectomy	RCT	d	Placebo	30	IM	Onabotulinum toxin type A-Botox®	40 for each pectoralis	Significant pain reduction in VAS score (p < .05)	-
(78)	Post-mastectomy	RS	na	Controls	48	IM	Botulinum toxin type A	100	Significant reduction pain in postoperative (*p* < 0.0001), during initial (*P* = 1.6 × 10(6)) and final expansion (*p* = 0.009)	-

PS, prospective study; RCT, randomized controlled trials; RS, retrospective study; s, single; d, double; na, not applicable; SC, subcutaneous; IM, intramuscular; *term as originally reported in the study; U, units; VAS, visual analog scale; NRS, numeric rating scale; -, not reported.

The prevalence of chronic post-surgical pain in cancer patients ranges from 20 to 70% according to different studies. Chronic post-surgical pain pathophysiology is likely to include both peripheral and sensitization mechanisms (80, 81).

A prospective study on 48 post-mastectomy patients demonstrated that BoNT/A (100 U) administration in the pectoralis major, serratus anterior, and rectus abdominis muscle followed by immediate insertion of tissue expander leads to a significant reduction of post-operative pain (*p* < 0.0001) and pain during both initial and final expansion (*p* = 0.009), greater volume of expansion per session (*p* = 0.010), reduced number of expansion sessions (*p* = 0.025), and lower narcotic use compared to standard procedures (*p*= 0.012) (78).

Similarly, a prospective RCT evaluating BoNT/A in expander-based breast reconstruction (40 U into the pectoralis major muscle), demonstrated a reduction in the use of oxycodone (*p* < 0.0001) and diazepam (*p* < 0.0001) and an increase in the expansion volume per visit in the active group compared to placebo (*p* < 0.05) (77).

BoNT/A (doses up to 100 U, intramuscularly or subcutaneously) proved effective in reducing pain and improving quality of life in eight out of 12 female cancer patients who had surgery or radiation for local cancer and failed >2 analgesic treatments (76) (Table 7).

### Complex Regional Pain Syndrome (CRPS)

CRPS is characterized by disabling chronic-relapsing burning pain, vasomotor changes, and occasionally trophic or motor function changes (82).

BoNT/A administration in muscular trigger points was reported to be effective in CRPS (83) but this finding was not confirmed in a larger prospective RCT on 14 individuals delivering BoNT/A into the allodynic skin areas (84). Caroll et al. randomly treated nine patients with refractory CRPS using standard lumbar sympathetic block (LSB) with bupivacaine (0.5%) or LSB with bupivacaine (0.5%) + BoNT/A (75 U) and found a significantly lower rate of pain return (*p* < 0.02) and greater reduction in pain intensity (*p* < 0.0001) in those receiving BoNT/A compared with local anesthetic alone (85).

In a retrospective, uncontrolled study, the EMG-guided administration of 100 U of BoNT/A (10–20 U per pain site) to 37 patients with severe local pain at baseline induced a significant pain reduction (mean pain score from 8.2 to 4.5; *p* < 0.001) in almost all individuals (97%) (86). Lumbar sympathetic block with levobupivacaine 0.25% 5 mL plus botulinum toxin type B 5,000 IU under fluoroscopic guidance was associated, 2 months later, to a meaningful reduction of pain intensity, allodynia, Leeds assessment of neuropathic symptoms, skin coldness and discoloration, and tissue swelling (87). The studies are summarized in Table 8.

**Table 8:** Studies on the use of botulinum toxin in CRPS.

**References**	**Study type**	**Blinding**	**Comparator**	**Pts n°**	**Injection route/site**	Toxin type*	**Dose (U)**	**Major findings**	**AEs**
(87)	Case report	na	na	2	Lumbar sympathetic block	Botulinum toxin type B-Myobloc®, Solstice Neurosciences, USA	5,000	Reduction in VAS score and CRPS symptoms	Nausea and vomiting (1 pt)
(83)	Cases series	na	na	2	IM	Botulinum toxin type A	20 for site (total 200)	Reduced pain and distal allodynia	-
(86)	RS	na	na	37	IM	Botulinum toxin type A	100	Pain reduction in 97% of patients	Transient neck drop (1 pt)
(84)	RCT + OL	d	Placebo	8+6	ID, SC	Onabotulinum toxin type A-Botox®	40–200	Ineffective	Poorly tolerated
(85)	RCT	d	0.5% bupivacaine	9	Sympathetic block	Botulinum toxin type A	75	Longer duration of pain reduction (71 vs. 10 days; *p* < 0.02). Significant VAS core reduction (*p* < 0.0001)	Nausea and vomiting (1 pt

CRPS, Complex regional pain syndrome; RCT, randomized controlled trials; RS, retrospective study; OL, Open label; d, double; na, not applicable; SC, subcutaneous; IM, intramuscular; ID, intradermal; *term as originally reported in the study; U, units; VAS, visual analog scale; -, not reported.

### Piriformis Syndrome

Piriformis syndrome is caused by the entrapment of the sciatic nerve by the piriformis muscle and accounts to up 8% of sciatic pain. The diagnosis of piriformis syndrome is sometimes challenging due to clinical overlap with low back and buttock pain. A single RCT on 56 patients treated with physical therapy and allocated to BoNT/A A (300 U) or placebo revealed a more marked reduction in VAS score, compared with placebo, at 2, 4, 6, 8, 10, and 12 weeks post-injection (*P* < 0.0001) (Table 9) (88).

**Table 9:** Studies on the use of botulinum toxin in Piriformis syndrome.

**References**	**Study type**	**Blinding**	**Comparator**	**Pts n°**	**Injection route/site**	Toxin type*	**Dose (U)**	**Major findings**	**AEs**
(88)	RCT	d	Placebo	56	IM	Incobotulinum toxin type A	300	Significant VAS score reduction at weeks 2,4,6,8, and 12 post-injection (*p* < 0.0001)	Injection site pain, stiff neck, wobbly neck, flu-like symptoms

RCT, randomized controlled trials; d, double; *term as originally reported in the study; U, units; VAS, visual analog scale; -, not reported.

### Phantom Limb Pain (PLP)

The long-term treatment of PLP using BoNT/A administration (4 × 25 U, quarterly) in the stump muscles of a lower limb amputee led to almost complete pain freedom (89).

A prospective RCT on 14 patients randomized to receive BoNT/A (250–300 U) or lidocaine plus depomedrol at the focal tender point demonstrated that both treatments were equally effective in PLP relief (Table 10) (90).

**Table 10:** Studies on the use of botulinum toxin in PLP.

**References**	**Study type**	**Blinding**	**Comparator**	**Pts n°**	**Injection route/site**	Toxin type*	**Dose (U)**	**Major findings**	**AEs**
(90)	Case report	na	na	1	IM	Onabotulinum toxin type A-Botox®	100 (4 injections performed every 3 months)	Almost complete pain-freedom	-
(89)	RCT	d	Lidocaine/Depomedrol	14	IM/cutaneous/SC	Onabotulinum toxin type A-Botox®	50 for sides	Not superior to placebo	-

PLP, Phantom Limb Pain; RCT, randomized controlled trials; d, double; na, not applicable; *term as originally reported in the study; IM, intramuscular; SC, subcutaneous; U, units; -, not reported.

### Spinal Cord Injury (SCI)

Pain often complicates SCI. Neuropathic pain after SCI is generally severe, refractory to treatment and persistent over time, reducing quality of life and interfering with cognitive, emotional, and physical functioning. Its prevalence rate ranges between 75 and 81% (91, 92).

A few case reports described a notable VAS score improvement of SCI-related neuropathic pain and allodynia using BoNT/A at a dose ranging from 80 to 200 U, documenting also a quite rapid onset of the clinical benefit and a long-lasting effect (>3 months) (93, 94). These promising findings were confirmed by two RCTs. BoNT/A (200 U) subcutaneous administration into the painful area proved effective in a trial on 40 patients affected by SCI-associated neuropathic pain, exhibiting a statistically significant decrease in VAS at weeks 4 and 8 compared to the placebo group (95). Similar results were reported by another study including 44 patients which documented a greater efficacy of BoNT/A over placebo in decreasing the VAS score after weeks 4 and 8 post treatments (*p* < 0.01) and in improving quality of life (Table 11) (96).

**Table 11:** Studies on the use of botulinum toxin in SCI.

**References**	**Study type**	**Blinding**	**Comparator**	**Pts n°**	**Injection route/site**	Toxin type*	**Dose (U)**	**Major findings**	**AEs**
(94)	Case report	na	na	1	SC	Botulinum toxin type A	10	VAS decreased from 96 to 23	-
(93)	Case report	na	na	2	SC	Onabotulinum toxin type A-Botox®	20	Significant VAS score reduction for > 3 months	-
(95)	RCT	d	Placebo	40	SC	Botulinum toxin type A- BTX-A Meditoxin Medytox, Seoul, South Korea	200	Significant VAS score reduction at week 4 (*p* < 0.0001) and at week 8 (*p* = 0.0012).	-
(96)	RCT	d	Placebo	44	SC	Botulinum toxin type A- HengLi Botox, Lanzhou, Gansu, China	200	Significant VAS score reduction at week 4 and week 8 of treatment (*p* < 0.01)	-

SCI, spinal cord injury; RCT, randomized controlled trials; d, double; na, not applicable; SC, subcutaneous; *term as originally reported in the study; U, units; VAS, visual analog scale; -, not reported.

### Neurogenic Thoracic Outlet Syndrome (NTOS)

NTOS is a complex entity characterized by different neurovascular signs and symptoms involving the upper limb due to a compression of the brachial plexus trunks or cords, including nerves which comes from the C5-T1 spinal levels. According to some studies, BoNT may be useful to reduce NTOS symptoms in those patients who did not benefit from physical therapy (97–99) (Table 12).

**Table 12:** Studies on the use of botulinum toxin in NTOS.

**References**	**Study type**	**Blinding**	**Comparator**	**Pts n°**	**Injection route/site**	Toxin type*	**Dose (U)**	**Major findings**	**AEs**
(98)	Case report	na	na	1	IM	Botulinum toxin type A	30	Symptomatic relief	-
(99)	RCT	d	Placebo	38	IM	Botulinum toxin type A	75	VAS scores did not result in clinically or statistically significant improvements in pain (*P* = 0.36).	-

NTOS, neurogenic thoracic outlet syndrome; RCT, randomized controlled trials; d, double; na, not applicable; IM, intramuscular; *term as originally reported in the study; U, units; VAS, visual analog scale; -, not reported.

### Chronic Pelvic Pain Syndrome (CPPS)

CPPS is defined as “a chronic pain and inflammation in the pelvic organs lasting >6 months” (100). Its treatment includes behavioral interventions, physical therapy, medications, nerve blocks, neurostimulation techniques, surgical interventions, and alternative therapies. BoNT/A has also been considered in a multimodal treatment plan in selected cases, being able to act on the pelvic peripheral nerves through different mechanisms. Some studies have reported encouraging results as regards VAS score reduction (101–104) (Table 13).

**Table 13:** Studies on the use of botulinum toxin in CPPS.

**References**	**Study type**	**Blinding**	**Comparator**	**Pts n°**	**Injection route/site**	Toxin type*	**Dose (U)**	**Major findings**	**AEs**
(101)	Pilot study	na	na	12	IM	Botulinum toxin type A Botox®	40	VAS and Quality of life scores significantly improved (p = 0.01)	Influenza-like symptoms (2 pts)
(102)	Pilot study	na	na	29	IM	Botulinum toxin type A	100	Partial response rate on the GRA (*p* = 0.0002). Significant reduction in CPSI pain subdomain score (*p* = 0.05)	-
(103)	RCT	d	Placebo	60	Transurethral intraprostatic	Botulinum neurotoxin type A	100	Significant VAS score decrease and significant improvement in QoL scores (*p* < 0.05)	-
(104)	PS	na	na	43	Transurethral intraprostatic	Onabotulinum toxin type A		VAS −79 and −27.4% at 3 and 12 months (*p* < 0.0001)	-

CPPS, chronic pelvic pain syndrome; RCT, randomized controlled trials; PS, prospective study; d, double; na, not applicable; IM, intramuscular; ^*^term as originally reported in the study; U, units; GRA, Global Response Assessment; CPSI, Chronic Prostatitis Symptom Index; VAS, visual analog scale; -, not reported.

## Conclusions

The majority of the studies were performed using BoNT/A, two studies using BoNT/B. VAS score reduction was the primary endpoint in all the studies. A positive effect of BoNT/A on NP was documented in 19 out of 21 RCT studies. The only RCT performed with BoNT/B provided negative results. Negative results emerged in two RCTs, (one in PSP and one in CTS) (69, 73) while one RCT in CRPS was stopped due to low tolerability (84). The positive effects of BoNT/A on NP started after 4–8 weeks [after 1 week in TN (34, 35, 39)] and persisted up to 6 months after treatment (34, 37–42, 45, 60, 62, 68, 70, 71, 89, 90, 94). The duration of BoNT/A benefit was dependent on toxin dose, injection site, number and depth of injections in NP (40, 41) but not in TN (34, 37–42, 45). The effect of BoNT/B, associated with levobupivacaine, was positive only in two case reports (87); negative effect of BoNT/B was observed in a pilot studies on 20 patients (73).

In all the studies, BoNT/A had been used as a second line treatment in patients who had had previous pharmacological therapeutic failures. Prior unresponsiveness to standard of care did not affect patient's responsivity to BoNT/A. The overall tolerability of BoNT/A in the different clinical setting was good, and adverse events were usually transient and mild. No safety concerns emerged. The treatment of the face for trigeminal neuralgia or post-herpetic neuralgia was burdened by a greater number of side effects compared to limb or thorax districts due to potential facial asymmetry induced by the muscle relaxant effect of BoNT/A.

NP is a chronic, highly disabling condition caused by a lesion or disease of the somatosensory nervous system which affects from 1.5 to 6.9% of individuals aged 50–64 years (10, 11).

There is a stringent need for innovative and alternative NP treatments because the current standard of care—including antidepressants, anticonvulsants, opioids, topical capsaicin, and lidocaine as well as non-pharmacological approaches—is still unsatisfactory due to the low responder rate (<30% of patients), the frequency and severity of adverse events (encompassing dizziness, ataxia, nausea, vomiting, somnolence, and cutaneous rash), and the relevant proportion of patients with treatment discontinuation (30–50%) (11, 13).

Botulinum toxin could represent a promising therapeutic tool for NP for its documented efficacy and tolerability in a wide range of NP conditions. BoNT/A is the toxin most extensively studied, having being investigated in 21 RCTs. BoNT/A seems helpful in particular in TN, PHN, PDN, occipital neuralgia, post-surgical pain and in SCI-related pain. However, the quality of evidence is low overall due to the paucity of RCT in some NP types, the small number of patients studied and methodological heterogeneities. One major limitation is the use of different toxin serotypes and preparations which hampers the comparison of studies' results. Most studies specified the use of the common BoNT/A brand (Botox®) (27, 29, 32, 34, 36, 40, 43, 44, 52, 55, 67–71, 74, 77, 84, 89, 90, 93, 101) or other BoNT/A compounds (e.g., HengLi®, Meditoxin®, Disport®) (33, 38, 39, 41, 50, 62, 72, 94, 96), but, in many cases, no specification of the BoNT/A serotype was provided (27, 31, 35, 37, 42, 47, 51, 53, 54, 60, 61, 66, 75, 78, 83, 85, 86, 94, 102–104). Furthermore, two studies were performed with botulinum toxin type B (73, 87) and two with incobotulinum toxin type A (76, 88).

No major safety issue emerged in the studies reported in the present review. Adverse events were rated as mild or moderate and included local skin reaction (swelling), pain at the injection site, muscles weakness, flu symptoms, nausea, and vomiting. However, there is a need for a specific evaluation of this aspect in human trials as, at present, only data on experimental animal models have been provided (105, 106).

The cost of the toxin and the need of specific injection expertise may represent a restriction for its widespread use.

Bearing in mind these limitations, we deem that the use of botulinum toxin should be carefully considered in patients with NP not responsive to current standard of care and to avoid undesired adverse events and safety concerns. BoNT could also reduce the use of surgical or invasive procedures, often applied in patients refractory to common therapeutic strategies.

Larger and specifically designed RCTs are awaited to confirm efficacy and tolerability of BoNT and also to provide standardized treatment models for the different types of NP, systematically specifying serotypes, doses, treatment sites, and the depth and number of injections. Future researches are also expected to ascertain the proportion of patients developing anti-toxin antibodies during prolonged treatment, evaluate the risk of systemic effects after local delivery and appraise the safety of the botulinum toxin in the elderly and in fragile individuals.

## Author Contributions

GE and LF equally contributed to the review of the literature and to the draft of the article. PB contributed to the draft and revision of the article. All authors contributed to the article and approved the submitted version.

## Conflict of Interest

GE has received travel grants and honoraria from Eli-Lilly, Novartis, and New Penta. LF has received travel grants and honoraria from TEVA, Eli-Lilly, and Novartis. PB has received travel grants or research support and honoraria or both for lecturing and being a consultant and scientific advisor for Merck, Lusofarmaco, Bayer, TEVA, Novartis, Eli-Lilly, Visufarma, and Assosalute.
